# 27-Hydroxycholesterol, cognition, and brain imaging markers in the FINGER randomized controlled trial

**DOI:** 10.1186/s13195-021-00790-y

**Published:** 2021-03-06

**Authors:** Anna Sandebring-Matton, Julen Goikolea, Ingemar Björkhem, Laura Paternain, Nina Kemppainen, Tiina Laatikainen, Tiia Ngandu, Juha Rinne, Hilkka Soininen, Angel Cedazo-Minguez, Alina Solomon, Miia Kivipelto

**Affiliations:** 1 Division of Neurogeriatrics, Center for Alzheimer Research, NVS, Karolinska Institutet, Stockholm, Sweden; 2 Division of Clinical Geriatrics, Center for Alzheimer Research, NVS, Karolinska Institutet, Stockholm, Sweden; 3 Division of Clinical Chemistry, Department of Laboratory Medicine, Karolinska University Hospital, Stockholm, Sweden; 4 Division of Clinical Neurosciences, Turku University Hospital, Turku, Finland; 5 Turku PET Centre, University of Turku and Turku University Hospital, Turku, Finland; 6 Institute of Public Health and Clinical Nutrition, University of Eastern Finland, Kuopio, Finland; 7 Joint Municipal Authority for North Karelia Social and Health Services, Joensuu, Finland; 8 Public Health Promotion Unit, Department of Public Health Solutions, Finnish Institute for Health and Welfare, Helsinki, Finland; 9 Institute of Clinical Medicine/Neurology, University of Eastern Finland, Kuopio, Finland; 10 Neurocenter, Neurology Kuopio University Hospital, Kuopio, Finland; 11 Ageing Epidemiology (AGE) Research Unit, School of Public Health, Imperial College London, London, UK; 12 Theme Aging, Karolinska University Hospital, Stockholm, Sweden

**Keywords:** Cholesterol metabolism, 27-Hydroxycholesterol, Biomarkers, Dementia, Multimodal intervention

## Abstract

**Background:**

27-Hydroxycholesterol (27-OH), the main circulating oxysterol in humans and the potential missing link between peripheral hypercholesterolemia and Alzheimer’s disease (AD), has not been investigated previously in relation to cognition and neuroimaging markers in the context of preventive interventions.

**Methods:**

The 2-year Finnish Geriatric Intervention Study to Prevent Cognitive Impairment and Disability (FINGER) included older individuals (60–77 years) at increased risk for dementia but without dementia or substantial cognitive impairment from the general population. Participants were randomized to a multidomain intervention (diet, exercise, cognitive training, and vascular risk management) or control group (general health advice) in a 1:1 ratio. Outcome assessors were masked to group allocation. This FINGER exploratory sub-study included 47 participants with measures of 27-OH, cognition, brain MRI, brain FDG-PET, and PiB-PET. Linear regression models were used to assess the cross-sectional and longitudinal associations between 27-OH, cognition, and neuroimaging markers, considering several potential confounders/intervention effect modifiers.

**Results:**

27-OH reduction during the intervention was associated with improvement in cognition (especially memory). This was not observed in the control group. The intervention reduced 27-OH particularly in individuals with the highest 27-OH levels and younger age. No associations were found between changes in 27-OH levels and neuroimaging markers. However, at baseline, a higher 27-OH was associated with lower total gray matter and hippocampal volume, and lower cognitive scores. These associations were unaffected by total cholesterol levels. While sex seemed to influence associations at baseline, it did not affect longitudinal associations.

**Conclusion:**

27-OH appears to be a marker not only for dementia/AD risk, but also for monitoring the effects of preventive interventions on cholesterol metabolism.

**Trial registration:**

ClinicalTrials.gov, NCT01041989. Registered on 4 January 2010

## Background

Alzheimer disease (AD) is the most common cause of dementia and considered a multifactorial disorder with a complex etiology [1, 2]. Lipid metabolism plays an important role in AD [3]. For example, brain cholesterol can affect amyloid precursor protein (APP) processing and amyloid deposition [4], as well as tau phosphorylation [5]. Elevated circulating cholesterol especially in midlife has been linked to increased risk of dementia and AD in epidemiological studies [6–8], via vascular-related pathways (e.g., atherosclerosis, stroke, and/or reduced brain blood flow [9]) or potential links to brain amyloid deposition [10, 11]. The association between altered lipid composition and aging, together with the recent progress of lipid research in AD, has led to an increased interest in studying lipid profiles and in particular cholesterol metabolites as fluid biomarkers of AD [12, 13].

The blood-brain barrier separates brain and peripheral cholesterol but allows the flux of its oxidized forms, oxysterols, where 27-hydroxycholesterol (27-OH) is one of the most abundant in the periphery [14]. Oxysterols affect a range of cellular functions and influence multiple physiological processes such as cholesterol metabolism, membrane fluidity, and intracellular signaling pathways. Hence, oxysterols play important roles in pathological conditions such as atherosclerosis, cancer, type 2 diabetes, and neurodegenerative disorders including AD [15–19]. 27-OH is derived from cholesterol oxidation by CYP27A1 which is expressed by most organs and tissues in humans [20]. High cholesterol in the periphery is associated with high levels of 27-OH in the circulation and thus increased transport of 27-OH into the brain. This transport is also dependent on the high rate of metabolism of 27-OH in the brain as well as by the integrity of the blood-brain barrier [21, 22]. Clinical studies have shown that the levels of 27-OH are increased in both cerebrospinal fluid [22–24] and brain tissue [25, 26] of AD patients compared to controls. Further, associations between reduced cognitive performance and high serum levels of 27-OH or ratio of 27-OH to cholesterol have been described in mild cognitive impairment [27] and older ApoE4 carriers [28].

Preclinical studies have pointed out several mechanisms underlying the effects of increased 27-OH on brain function. Notably, CYP27A1-overexpressing mice that produce increased levels of 27-OH develop memory impairment due to, e.g., reduction in neuronal glucose uptake [29], impaired neuronal branching and reduced synaptic density [30], inflammation and impairment of the brain renin-angiotensinogen system [24]. Taken together, the data from preclinical and clinical studies have led to the hypothesis that 27-OH may link AD and peripheral hypercholesterolemia [16].

While the role of 27-OH in AD has been studied in animal models and memory clinic patients in relation to cognitive measures and ApoE4 risk allele, no studies have so far investigated 27-OH in the context of preventive interventions. The Finnish Geriatric Intervention Study to Prevent Cognitive Impairment and Disability (FINGER) was a 2-year randomized controlled trial that reported beneficial effects on cognition in an at-risk older general population for a multimodal lifestyle/vascular intervention (combining cardiovascular risk management, diet, exercise, and cognitive training) versus regular health advice control [31]. In the current exploratory study, 27-OH was measured in serum samples from a subset of FINGER participants (*n* = 47), in order to investigate (1) whether the 2-year multidomain lifestyle/vascular intervention had an impact on the 27-OH levels and (2) whether the change in 27-OH during the intervention was related to the change in cognition and dementia-related neuroimaging markers (i.e., gray matter volume, cortical thickness and white matter lesions (WML) on MRI, glucose uptake on FDG-PET, and amyloid load on PiB-PET). In addition, we also investigated the associations of 27-OH with cognition and brain imaging markers at baseline.

## Material and methods

### Study participants

**Fig. 1:**
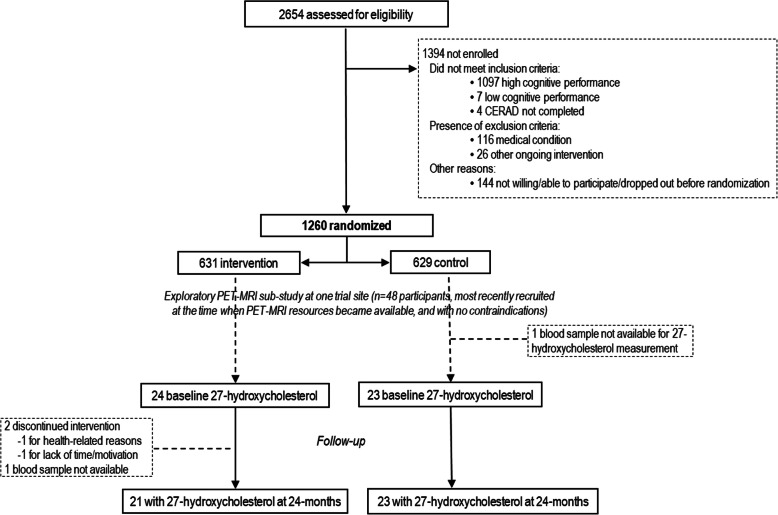
CONSORT diagram of the FINGER exploratory 27-hydroxycholesterol sub-study. CERAD, Consortium to Establish a Registry for Alzheimer’s Disease

This exploratory sub-study included 47 FINGER trial participants (21 women and 26 men, mean age 71 ± 5.1 years) who underwent both PET and MRI imaging at one of the six trial sites (Turku in southwestern Finland) [32] with serum samples available for 27-hydroxycholesterol measurements (Fig. 1). They were selected from the most recently recruited trial participants at the time when MRI/PET resources became available and if there were no contraindications. The demographic, clinical, and cognitive characteristics of these participants have been previously described in detail [32, 33], i.e., they were not different from the rest of the Turku cohort or the rest of the FINGER participants, except for being slightly older (mean 70.8 ± 5 years vs 69.3 ± 4.7 years for the rest of FINGER participants, due to a later initiation of the recruitment in Turku).

The FINGER population characteristics [34], trial protocol [35], primary findings [31], and neuroimaging sub-study [32, 36] have been previously published. In brief, between September 7, 2009, and November 24, 2011, 2654 individuals were screened and 1260 participants aged 60–77 years from the general population were recruited based on the Cardiovascular Risk Factors, Aging and Dementia (CAIDE) risk score [37] of 6 points or higher, and cognitive performance at the mean level or slightly lower than expected for age according to Finnish population norms for the Consortium to Establish a Registry for Alzheimer’s Disease (CERAD) [38]. Individuals were excluded if they had dementia or substantial cognitive impairment, any conditions affecting safe participation/cooperation, or ongoing participation in any other clinical trial.

### Intervention

Participants were randomized 1:1 into the intensive multidomain lifestyle intervention or control group. Outcome assessors were blinded to group allocation, and they were not involved in the intervention activities. The control group received regular health advice. As previously described in detail, the intervention group received nutrition guidance (individual and group sessions with the study nutritionists), physical exercise program at the gym supervised by study physiotherapists, cognitive training (individual computer-based training and group sessions led by study psychologists), and management of metabolic and vascular risk factors [35]. The intervention duration was 2 years.

### Cognitive outcomes

Standard cognitive testing (an extended version of the neuropsychological test battery (NTB) [39]) was conducted for all FINGER participants at the baseline and 12-month and 24-month visits. Pre-specified cognitive outcomes included the NTB total score (composite score based on all 14 tests, calculated as *Z*-scores standardized to the baseline mean and SD), NTB memory, executive functioning, and processing speed domain *Z*-scores calculated as described previously [35]. Higher scores indicated better performance.

### MRI and PET imaging

The subsample of FINGER participants from the Turku site underwent structural 3T MRI (Philips Ingenuity TF PET/MR, Amsterdam, the Netherlands), ^18^F-FDG dynamic PET scan (GE Advance PET scanner in the 3D scanning mode, General Electric Medical Systems, Milwaukee, WI, USA), and ^11^C-Pittsburgh compound B (PiB)-PET scan (Philips Ingenuity TF PET/MR, Amsterdam, the Netherlands) in connection to both the baseline and 24-month visits. The detailed imaging protocols have been previously published [32, 33].

MRI images were quality checked and read for any abnormalities. Regional brain volumes and cortical thickness were measured using the Freesurfer image analysis suite (version 5.0.3). Brain volumes were normalized to the total intracranial volume to adjust for head size. A measure of cortical thickness in AD signature regions was calculated as the average of cortical thickness in the entorhinal, inferior temporal, middle temporal, and fusiform regions [40]. White matter lesion (WML) volume was measured through the segmentation of white matter hyperintensities on T1 and FLAIR images [41].

For FDG-PET, a dose of ^18^F-FDG 3.7 MBq/kg was injected into an antecubital vein as a bolus with a mean dose of 459 MBq (SD 85 MBq) and flushed with saline. For amyloid PET, on average, 406.3 MBq (SD 107.7 MBq) of ^11^C-PiB-PET was injected intravenously. The scans were quantitatively assessed with the automated region of interest analysis. Composite measures were also calculated: for ^18^F-FDG uptake, the average across the prefrontal, parietal, lateral temporal, precuneus, anterior cingulate, and posterior cingulate cortex; for amyloid deposition, the average across the prefrontal, parietal, lateral temporal, precuneus, anterior cingulate, and posterior cingulate regions of interest. In addition, the amyloid PET scans were visually interpreted by two experienced readers and judged as visually positive or negative after a 2-party consensus agreement.

### Blood measurements

Fasting venous blood samples collected at the baseline and 24-month visits were used in the present study. Serum samples were analyzed for 27-hydroxycholesterol assayed by isotope dilution mass spectrometry using deuterium-labeled internal standards [42]. For this study, total cholesterol was also assayed by isotope dilution mass spectrometry according to a previously described protocol [43].

### Statistical analysis

For comparisons of the baseline characteristics between the intervention and control groups, *t* test or *χ*^2^ test was used as appropriate. Cross-sectional and longitudinal associations between 27-OH and other variables of interest were investigated using linear regression models, and zero-skewness log transformation was applied to skewed variables.

Change over time in 27-OH, cognitive, MRI, FDG-PET, and PIB-PET measures was calculated as the difference between 24-month and baseline values. To study the intervention effect on 27-OH, a linear regression model was conducted with change in 27-OH as an dependent variable and randomization group as an independent variable. To study whether the changes in cognitive, MRI, FDG-PET, and PIB-PET measures (dependent variables) were related to 27-OH, the linear regression models included the randomization group, change in 27-OH, and their interaction. Potential relevant confounders (age, sex, ApoE4 carrier status, lipid-lowering drugs, or total cholesterol change) were also adjusted for additional models.

Analyses of baseline cognitive, MRI, FDG-PET, and PIB-PET measures (as dependent variables) in relation to baseline 27-OH were adjusted for age, self-reported lipid-lowering drugs (and/or diabetes medication for FDG-PET analyses), education (for cognitive measures), and time between blood sample collection and brain scan (for MRI, FDG-PET, and PIB-PET measures) (model 1). Additional adjustments for total cholesterol (model 2) and sex (model 3) were also applied.

The level of significance was set to *p* < .05 in all analyses, and the Stata software, version 14 (StataCorp), was used.

## Results

### Population characteristics

Baseline characteristics of the intervention and control groups are listed in Table 1. Demographic, cognitive, MRI, and PiB-PET measures showed no differences between the groups. The intervention group had significantly lower mean baseline ^18^F-FDG-PET uptake in the prefrontal and parietal cortex, as well as lower FDG-PET cortical composite measure compared with the control group.

**Table 1:** Baseline characteristics of the control and intervention groups

		Number	Control, mean (SD)	Number	Intervention, mean (SD)	*p*
**Demographic characteristics**						
Sex, % men/women		11/12	47.8/52.2	15/9	62.5/37.5	0.30
Age, years		23	70.1 (4.7)	24	71.2 (5.4)	0.46
Years of education		23	9.3 (2.5)	24	9.1 (2.4)	0.76
Smokers, %, no/yes		20/2	90.9/9.1	23/1	95.8/4.2	0.51
ApoE4 carrier, %, no/yes		16/6	72.7/27.3	16/8	66.7/33.3	0.70
*ApoE* allele frequencies	e2/e3	1		1		
	e2/e4	0		2		
	e3/e3	15		15		
	e3/e4	5		6		
	e4/e4	1		0		
**Serum measurements**						
27-OH, ng/ml		23	176.48 (48.39)	24	182.42 (49.28)	0.68
Total cholesterol, mmol/l		23	4.87 (1.06)	24	4.99 (1.00)	0.69
**Cognition**						
NTB total composite		23	0.01 (0.54)	24	−0.04 (0.53)	0.75
NTB memory		23	0.02 (0.64)	24	−0.14 (0.52)	0.35
NTB processing speed		23	0.14 (0.74)	24	−0.03 (1.05)	0.53
NTB executive function		23	−0.09 (0.56)	24	0.07 (0.58)	0.35
**MRI**						
*Total gray matter volume, ml		23	568.35 (59.35)	24	582.86 (51.80)	0.38
*Total hippocampal volume, ml		23	7.20 (0.94)	24	7.73 (0.99)	0.07
AD signature cortical thickness, mm		20	2.73 (0.13)	19	2.72 (0.10)	0.70
*White matter lesion volume, ml		23	12.49 (16.49)	23	14.06 (16.18)	0.75
Total intracranial volume, ml		23	1540.64 (257.41)	24	1556.66 (252.14)	0.83
**FDG-PET**						
Cortical composite		23	1.23 (0.07)	22	1.19 (0.08)	**0.03**
Prefrontal cortex		23	1.35 (0.08)	22	1.27 (0.09)	**0.01**
Parietal cortex		23	1.24 (0.10)	22	1 19 (0.08)	**0.05**
Lateral temporal cortex		23	1.02 (0.05)	22	0.99 (0.05)	0.10
Precuneus		23	1.38 (0.12)	22	1.33 (0.10)	0.13
Anterior cingulate gyrus		23	1.12 (0.08)	22	1.08 (0.10)	0.14
Posterior cingulate gyrus		23	1.29 (0.09)	22	1.24 (0.11)	0.09
Medial temporal cortex		23	0.79 (0.05)	22	0.78 (0.06)	0.48
Lateral occipital cortex		23	1.17 (0.08)	22	1.15 (0.09)	0.37
Striatum		23	1.19 (0.10)	22	1.13 (0.10)	0.07
Cerebellum		23	1.02 (0.01)	22	1.02 (0.01)	0.66
**PiB PET**						
Cortical composite		23	1.57 (0.37)	24	1.46 (0.38)	0.34

Values are means ± SD unless otherwise specified. Between-group differences were analyzed with the chi-square and *t* tests as appropriate. *p* value considered significant (marked in bold) if < 0.05. Cognitive scores are mean values of *Z*-scores of the cognitive tests included in each cognitive outcome, where higher scores indicate better performance. *Brain volumes are shown unadjusted; corresponding *p* values are shown from analyses with values adjusted for total intracranial volume and zero-skewness log-transformed as appropriate. AD signature cortical thickness was calculated as the average of cortical thickness in the entorhinal, inferior temporal, middle temporal, and fusiform regions. The composite scores for FDG-PET and PiB-PET were determined as the average of the prefrontal, parietal, lateral temporal, anterior cingulate, posterior cingulate, and precuneus ROIs

*ApoE4*, apolipoprotein E4; *NTB*, neuropsychological test battery; *MRI*, magnetic resonance imaging; *AD*, Alzheimer’s disease; *FDG-PET*, 2-deoxy-2fluorine-18fluoro-d-glucose positron emission tomography; *PiB*, 11 C-Pittsburgh compound B

### Intervention effects on change in serum 27-OH levels

Mean 27-OH levels (± SD) decreased during 2 years in the intervention group (− 2.8 ± 55.6 ng/ml) and increased in the control group (+ 16.1 ± 55.3 ng/ml), although the change was not significantly different between groups (*p* = 0.26).

**Fig. 2:**
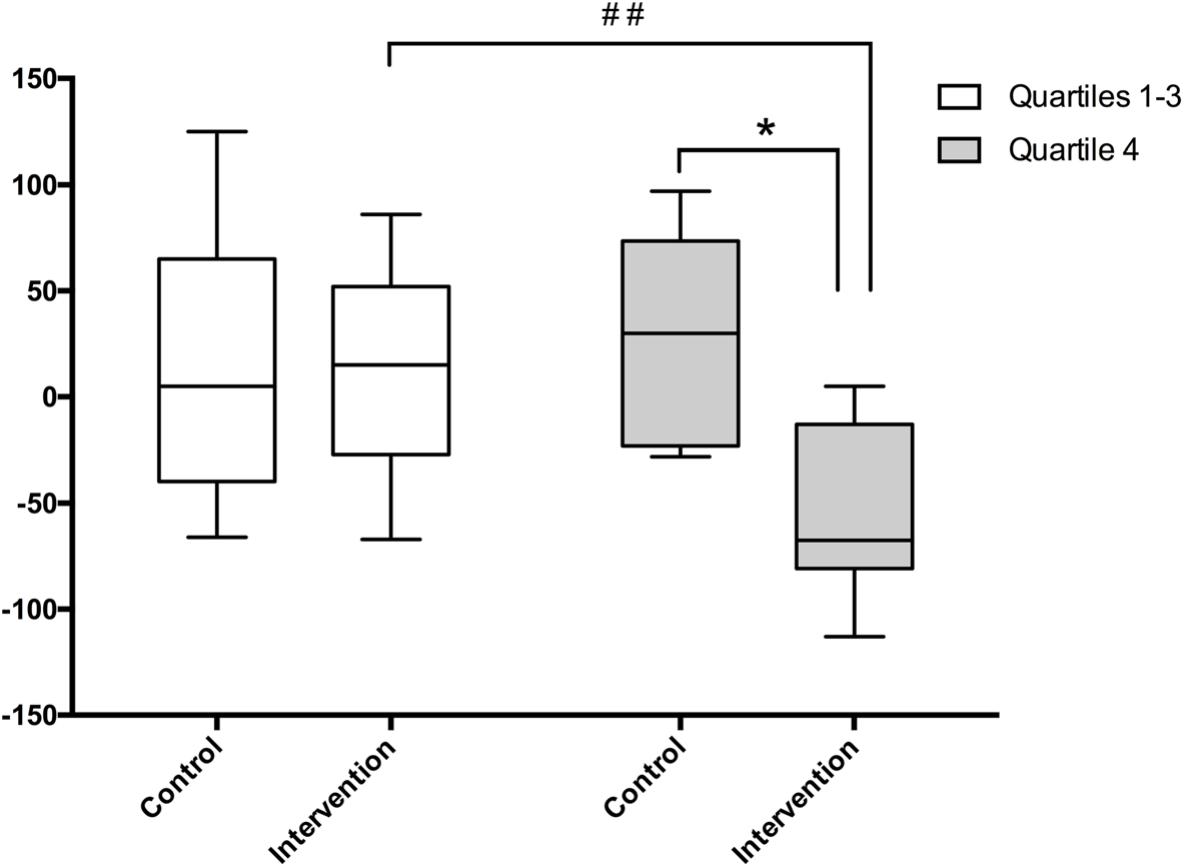
FINGER intervention effects on the change in 27-OH. Participants were divided into two groups according to their baseline 27-OH levels: the lowest 75% (quartiles 1–3, white boxplots) and the highest 25% (quartile 4, gray boxplots). The graph shows the mean change in 27-OH levels (ng/ml) between the 2-year and baseline measurements. Mann-Whitney non-parametric *U* test was used to analyze the differences between quartiles 1–3 and 4 of the intervention group (^#^^#^< 0.01) and between quartile 4 of the control and intervention group (*p < 0.05)

We conducted additional exploratory sub-group analyses of the potential effect of high versus low baseline 27-OH on change over time in 27-OH. The highest (4th) quartile of baseline 27-OH (*n* = 11 participants) was compared with quartiles 1–3 (*n* = 33 participants) since there is currently no established cutoff for high 27-OH (Fig. 2). In the intervention group, mean 27-OH change was + 18 ng/ml in quartiles 1–3, and − 55 ng/ml in quartile 4 (*p* = 0.004 for comparison between quartile 4 and quartiles 1–3). In the control group, the mean 27-OH change was + 13 ng/ml in quartiles 1–3, and + 26 ng/ml in quartile 4. The difference between the change in 27-OH in quartile 4 in the intervention versus the control group was significant (*p* = 0.03), suggesting that the intervention was most efficient in reducing the highest 27-OH levels.

We also investigated if the intervention effect on 27-OH was affected by age at baseline, sex, ApoE4 carrier status, lipid-lowering medication during the intervention, or change in total cholesterol level (Table 2). 27-OH decreased significantly more in the intervention group vs the control group among younger, but not older participants (randomization group × age interaction, *p* = 0.01). Sex, ApoE4 carrier status, lipid-lowering medication, or total cholesterol change had no significant impact on the intervention effect on 27-OH.

**Table 2:** Intervention effect on 27-OH and impact of age, sex, ApoE4 carrier status, lipid-lowering medication, and change in total cholesterol

Factor	Difference between intervention and control		Randomization group x Factor interaction, *p*
	N*	*β* (*p*)	
Age at baseline	< 70 years, *N* = 22	−0.51 (**0.02**)	**0.01**
	≥70 years, N = 22	0.21 (0.34)	
Sex	Women, *N* = 20	− 0.06 (0.81)	0.52
	Men, *N* = 24	−0.24 (0.26)	
ApoE4 carrier status	No, *N* = 29	−0.13 (0.53)	0.96
	Yes, *N* = 14	−0.13 (0.65)	
Lipid-lowering medication	No, *N* = 22	−0.13 (0.57)	0.77
	Yes, *N* = 22	−0.21 (0.34)	
Change in total cholesterol	Decrease, *N* = 28	−0.19 (0.33)	0.43
	No change/increase, *N* = 16	−0.18 (0.50)	

Values are standardized *β* coefficients (*p* values) from linear regression models with change in 27-OH (difference between 2-year and baseline values) as a dependent variable. Differences between the intervention and control groups are shown from models with randomization group as an independent variable and stratified by each factor (age at baseline, sex, ApoE4 carrier status, lipid-lowering medication, or change in total cholesterol). *p* value for interaction shown from models including randomization group, factor (as continuous variable for age or total cholesterol change), and the randomization group *x* factor interaction. *p* values considered significant (bold) if < 0.05. *N: participants with available data from both baseline and 2-year follow-up for each analysis

### Longitudinal associations of serum 27-OH levels with cognition and neuroimaging markers

Table 3 shows associations between the change in 27-OH levels and the change in cognitive scores and brain imaging measures. Overall, change in 27-OH was not associated with the change in cognition or neuroimaging measures. However, there was a significant interaction between the randomization group and change in 27-OH in relation to the change in NTB memory score (*p* = 0.02). A reduction in 27-OH was associated with improvement in NTB memory score in the intervention group (*β* = − 0.54, *p* = 0.01), but this association was not found in the control group (*β* = 0.12, *p* = 0.58). There was a similar pattern for change in NTB total composite and total gray matter volume, although the randomization group × 27-OH interactions showed only non-significant trends (*p* = 0.07 and *p* = 0.08, respectively). In stratified analyses, a reduction in 27-OH in the intervention group was significantly related to improvement in NTB total composite score (*β* = − 0.46, *p* = 0.04) and a trend for a less pronounced decline in total gray matter volume (*β* = − 0.39, *p* = 0.10). These associations were not found in the control group.

**Table 3:** Longitudinal associations between change in 27-OH and change in cognition and neuroimaging markers

	Control, *β* (*p*)	Intervention, *β* (*p*)	Interaction, *p*
**Change in cognition**			
NTB total composite	0.11 (0.62)	**− 0.46 (0.04)**	0.07
NTB memory	0.12 (0.58)	**− 0.54 (0.01)**	**0.02**
NTB processing speed	−0.04 (0.85)	0.24 (0.31)	0.41
NTB executive function	0.12 (0.59)	−0.31 (0.17)	0.18
**Change in MRI**			
Total gray matter volume	0.18 (0.45)	−0.39 (0.10)	0.08
Hippocampal volume	0.06 (0.79)	−0.28 (0.25)	0.30
AD signature cortical thickness	0.05 (0.84)	−0.20 (0.42)	0.50
White matter lesion volume	−0.05 (0.82)	−0.05 (0.85)	0.97
**Change in FDG-PET**			
Cortical composite	0.24 (0.32)	0.01 (0.97)	0.42
Prefrontal cortex	0.08 (0.74)	0.04 (0.89)	0.85
Parietal cortex	0.21 (0.36)	0.11 (0.70)	0.68
Lateral temporal cortex	0.20 (0.40)	−0.03 (0.91)	0.46
Precuneus	0.23 (0.33)	−0.04 (0.89)	0.41
Anterior cingulate gyrus	0.25 (0.29)	−0.05 (0.85)	0.34
Posterior cingulate gyrus	0.26 (0.27)	0.00 (0.99)	0.34
Medial temporal cortex	0.25 (0.30)	0.13 (0.64)	0.59
Lateral occipital cortex	0.23 (0.32)	−0.28 (0.29)	0.18
Striatum	0.38 (0.10)	−0.08 (0.77)	0.15
Cerebellum	−0.01 (0.97)	0.22 (0.41)	0.50
**Change in PIB-PET**			
Cortical composite	−0.04 (0.87)	−0.10 (0.69)	0.81

Values are standardized *β* coefficients (*p* values) from linear regression models with change in cognitive and neuroimaging measures (left column) as dependent variables and change in 27-OH as an independent variable. Change in all measures was calculated as the difference between 2-year and baseline values. Values for the control and intervention groups are shown from analyses stratified by the randomization group. *p* value for interaction is shown from models including change in 27-OH, randomization group, and their interaction. *p* value considered significant (bold) if < 0.05. AD signature cortical thickness was calculated as the average of cortical thickness in the entorhinal, inferior temporal, middle temporal, and fusiform regions. The changes in composite scores for FDG-PET and PiB-PET were determined as the average change of the prefrontal, parietal, lateral temporal, anterior cingulate, posterior cingulate, and precuneus ROIs

*NTB*, neuropsychological test battery; *MRI*, magnetic resonance imaging; *AD*, Alzheimer’s disease; *FDG-PET*, 2-deoxy-2-fluorine-18fluoro-d-glucose positron emission tomography; *PiB*, 11 C-Pittsburgh compound B

Additional adjustment for age at baseline, sex, lipid-lowering drugs, or change in total cholesterol did not change the observed associations (results not shown), except for the change in NTB memory score where the randomization group × 27-OH change interaction became a non-significant trend (*p* = 0.09) after adjusting for age.

### Baseline associations of serum 27-OH levels with cognition and neuroimaging markers

Association between 27-OH, cognition, and neuroimaging markers at baseline are shown in Table 4. Higher 27-OH was associated to lower NTB total composite (*β* = − 0.28, *p* = 0.04) and NTB memory scores (*β* = − 0.36, *p* = 0.01) in model 1. These associations remained significant after adjusting for total cholesterol levels (*β* = − 0.42, *p* = 0.01 for NTB total composite and *β* = − 0.59, *p* = 0.00 for NTB memory) (model 2). After further adjusting for sex (model 3), there was only a non-significant trend for association with NTB memory (*β* = − 0.27, *p* = 0.08), and the association with NTB total composite was no longer statistically significant (*β* = − 0.22, *p* = 0.12). No significant associations were found with processing speed or executive functioning scores in any of the models.

**Table 4:** Cross-sectional associations between 27-OH, cognition, and neuroimaging markers at baseline

	Model 1, *β* (*p*)	Model 2, *β* (*p*)	Model 3, *β* (*p*)
**Cognition**			
NTB total composite	**−0.28 (0.04)**	**−0.42 (0.01)**	−0.22 (0.12)
NTB memory	**−0.36 (0.01)**	**−0.59 (0.00)**	−0.27 (0.08)
NTB processing speed	−0.20 (0.20)	−0.27 (0.14)	−0.16 (0.34)
NTB executive function	−0.09 (0.53)	−0.08 (0.62)	−0.08 (0.57)
**MRI**			
Total gray matter volume	−0.27 (0.09)	**−0.45 (0.01)**	−0.08 (0.61)
Hippocampal volume	**−0.31 (0.04)**	**−0.48 (0.00)**	−0.14 (0.31)
AD signature cortical thickness	0.01 (0.98)	0.01 (0.96)	−0.15 (0.42)
White matter lesion volume	0.24 (0.13)	0.31 (0.10)	0.21 (0.22)
**FDG-PET**			
Cortical composite	−0.22 (0.16)	−0.27 (0.11)	−0.08 (0.66)
Prefrontal cortex	−0.03 (0.86)	−0.04 (0.82)	−0.08 (0.66)
Parietal cortex	−0.27 (0.09)	−0.30 (0.09)	−0.16 (0.38)
Lateral temporal cortex	−0.21 (0.21)	−0.27 (0.14)	−0.13 (0.48)
Precuneus	−0.28 (0.10)	−0.32 (0.09)	−0.11 (0.55)
Anterior cingulate gyrus	−0.17 (0.27)	−0.20 (0.24)	−0.09 (0.62)
Posterior cingulate gyrus	−0.20 (0.23)	−0.30 (0.08)	−0.02 (0.91)
Medial temporal cortex	−0.02 (0.90)	−0.14 (0.44)	−0.05 (0.80)
Lateral occipital cortex	−0.31 (0.07)	−0.29 (0.11)	**−0.40 (0.04)**
Striatum	−0.28 (0.10)	−0.26 (0.15)	−0.27 (0.16)
Cerebellum	0.00 (0.98)	−0.05 (0.81)	0.21 (0.25)
**PIB-PET**			
Cortical composite	−0.06 (0.73)	−0.08 (0.67)	−0.02 (0.91)

Values are standardized *β* coefficients (*p* values) from linear regression models with cognitive and neuroimaging measures (left column) as dependent variables and 27-OH as an independent variable. Model 1 was adjusted for age, self-reported lipid-lowering medication (and/or diabetes medication for FDG-PET measures), education (for cognitive measures), and time between blood sample collection and brain scan (for MRI, FDG- and PIB-PET measures). Model 2 was additionally adjusted for serum total cholesterol. Model 3 was additionally adjusted for sex. *p* values considered significant (bold) if < 0.05. AD signature cortical thickness was calculated as the average of cortical thickness in the entorhinal, inferior temporal, middle temporal, and fusiform regions. FDG-PET and PiB-PET cortical composite scores were calculated as the average across prefrontal, parietal, lateral temporal, precuneus, anterior cingulate, and posterior cingulate cortex

*NTB*, neuropsychological test battery; *MRI*, Magnetic Resonance Imaging; *AD*, Alzheimer’s disease; *FDG-PET*, 2-deoxy-2-fluorine-18fluoro-d-glucose Positron emission tomography; *PiB*, 11 C-Pittsburgh compound B

Higher 27-OH was related to lower hippocampal volume (*β* = − 0.31, *p* = 0.04), with a non-significant trend for lower total gray matter volume (*β* = − 0.27, *p* = 0.09) (model 1). These associations were significant after adjusting for total cholesterol (model 2) (*β* = − 0.48, *p* = 0.00 and *β* = − 0.45, *p* = 0.01, respectively), but not after additional adjustment for sex (model 3) (*β* = − 0.14, *p* = 0.31 and *β* = − 0.08, *p* = 0.61 respectively). No significant associations were found with WML volume or cortical thickness in AD signature areas.

There was a consistent pattern of association between higher 27-OH and lower ^18^F-FDG uptake on PET scans across several brain regions, although the associations did not reach statistical significance. Adjustment for ApoE4 carrier status did not influence the associations seen (data not shown).

Given the impact of sex on the regression models, and known sex differences in peripheral 27-OH levels, we also compared baseline characteristics between men and women. As expected, 27-OH was higher for men (194 ± 52 ng/ml) vs women (162 ± 38 ng/ml), *p* = 0.02. Total gray matter volume and hippocampal volume (adjusted for head size) were significantly larger in women compared to men (0.40 vs 0.36, *p* = 0.00 and 0.0054 vs 0.0046, *p* = 0.01). No differences were found for total cholesterol levels, cognitive, demographic, or other neuroimaging measures (results not shown).

## Discussion

Peripheral hypercholesterolemia and brain cholesterol metabolism are features known to influence both the risk and the progression of the disease in AD and other dementia-related disorders [16, 44]. 27-OH is a key link between the peripheral and brain cholesterol pools and associations between 27-OH, cognition and related biomarkers have been previously analyzed in observational studies [22, 24]. In this exploratory FINGER sub-study, we investigated for the first time the effect of a dementia preventive intervention on 27-OH, and its relation to the change in cognition and neuroimaging markers. A reduction in 27-OH during the multidomain lifestyle/vascular intervention was associated with improvement in cognition (especially memory, with a trend for overall cognitive performance). 27-OH reduction during the intervention also tended to be related to less decline in total gray matter volume. These associations were not observed in the control group, where 27-OH levels tended to increase over time. The multidomain lifestyle/vascular intervention seemed to reduce 27-OH particularly in individuals with the highest 27-OH levels at baseline. Thus, our findings suggest that benefits on cognition and structural brain changes may require more intensive modifications of an individual’s cholesterol metabolites. These findings are especially important given that the main FINGER trial reported no significant intervention benefits on total cholesterol [31]. 27-OH may be a more sensitive marker for monitoring the benefits of lifestyle/vascular interventions on cholesterol metabolism. Reduction in 27-OH may also represent one possible mechanism behind the previously reported intervention benefits on cognition [31].

Interestingly, age (but not sex, lipid-lowering medication, or change in total cholesterol) had a significant impact on the FINGER intervention effect on 27-OH. It is not fully clear why younger individuals had more intervention benefits on 27-OH reduction compared with older individuals. Participants in the highest 27-OH quartile at baseline tended to be younger (mean 68.4 vs 71.4 years for the lower 27-OH quartiles), which may at least partly explain this finding. Future studies on a larger sample are needed to understand more about this association.

We found no statistically significant longitudinal associations between change in 27-OH and change in other MRI, FDG-PET, or PiB-PET measures. FINGER participants were older individuals with vascular/lifestyle risk factors, but without substantial cognitive impairment or dementia, i.e., the preventive intervention was started early, before the occurrence of extensive brain pathology. Overall, changes in neuroimaging parameters in this population were relatively small during 2 years [36]. In addition, the multidomain lifestyle/vascular intervention was designed to target multiple risk factors and disease-related mechanisms simultaneously, i.e., it did not specifically target amyloid pathology. If 27-OH reduction is a possible mechanism for intervention benefits on cognition, this mechanism may not necessarily be related to amyloid accumulation. Ongoing extended FINGER follow-ups will provide further data on longer-term changes in neuroimaging markers and how they relate to changes in cholesterol metabolism.

Previous observational studies in patients with mild cognitive impairment and elderly ApoE4 carriers have reported associations between elevated 27-OH and poorer cognitive performance [27, 28]. The present study investigated for the first time serum 27-OH, cognition, and related neuroimaging markers in at-risk older individuals without dementia or substantial cognitive impairment. In cross-sectional analyses at baseline, higher levels of 27-OH were associated with lower scores on global cognition and memory, and lower total gray matter and hippocampal volumes. These associations were influenced by sex, possibly due to men having higher 27-OH compared with women [45], e.g., because of generally higher cholesterol levels and/or reduced rate of metabolism. Other possible explanations may be sex differences in BBB permeability [46] allowing a higher proportion of 27-OH to enter the brain in men [47] or other sex-related factors not accounted for in this study.

However, the associations between 27-OH, cognition, and MRI measures were not substantially affected by serum total cholesterol levels. Findings from the present study thus support the notion that 27-OH acts independently of cholesterol on brain functioning. Potential mechanisms have been described in in vitro and in vivo experiments in models with elevated 27-OH. Along with memory impairment, CYP27A1 tg mice have an upregulated brain renin-angiotensin system [24] and reduction in dendritic arborization, spine density of the hippocampus [30], and levels of long-term memory-related protein Arc (activity-regulated cytoskeleton-associated protein). Importantly, CYP27A1 knock-out mice show less memory impairment upon high-fat diet and restoration of cholesterol-decreased levels of Arc in comparison with wild-type controls [48], a finding suggesting that 27-OH is an important factor mediating the risk of cognitive impairment caused by dietary cholesterol. A previous study on cognitively impaired patients showed that high 27-OH levels in CSF were associated with reduced brain glucose uptake [29]. These patients did not have high cholesterol levels nor lipid-lowering medications, suggesting that the associations were independent of total cholesterol levels but rather related to a high cholesterol metabolic rate.

CYP27A1, the enzyme responsible for converting cholesterol to 27-OH, was recently explored as a new target for breast cancer adjuvant therapy. The approved drug anastrazole was shown to successfully inhibit CYP27A1 and thereby lower 27-OH levels without altering cholesterol levels in mice [49]. Pharmaceutical adjustment of oxysterols, and in particular 27-OH in combination with a lifestyle intervention, may be an attractive new avenue for dementia prevention. Given the important role of CYP27A1 for the formation of bile acids, altering the levels of 27-OH must however be monitored carefully.

In this exploratory FINGER sub-study, although there seemed to be a consistent pattern of association between higher serum 27-OH and lower brain glucose uptake, the associations did not reach statistical significance. This could be due to two main reasons: (i) the small sample size and thus limited statistical power of this study and (ii) FINGER included at-risk participants without dementia or substantial impairment, while a more substantial and widespread impact of 27-OH on glucose uptake in the brain may become more evident in later stages of disease progression. In the future, follow-up studies on larger cohorts including the prodromal AD stage would be helpful to further investigate the role of 27-OH on glucose brain uptake in disease progression.

27-OH levels were not significantly related to amyloid accumulation on PiB-PET scans at baseline in the present study. Previous experimental studies have provided conflicting data on amyloid and 27-OH, with 27-OH reported to have a slight inhibitory effect on APP secretion and amyloid production [50], but also to increase BACE1, APP, and Aβ levels [51]. No correlation was found between CSF levels of 27-OH and Aβ42, but with plasma 27-OH and CSF soluble APP [52]. The relationship between amyloid and 27-OH in at-risk states and early disease stages should be further investigated in larger cohorts.

The main strengths of the present study are the randomized controlled design, multidomain lifestyle/vascular intervention with high relevance for peripheral lipid metabolism, and comprehensive repeated assessments of 27-OH, cognition, and related neuroimaging markers during 2 years.

### Limitations

The main limitation is the small sample size affecting statistical power. Because all analyses are post hoc and no correction for multiple testing has been applied, findings should be considered exploratory and will need to be verified in further studies with larger sample sizes.

## Conclusions

In conclusion, a reduction in 27-OH during the multidomain lifestyle/vascular intervention was associated with improvement in cognition (especially memory). This was not observed in the control group. The intervention seemed to reduce 27-OH particularly in individuals with the highest 27-OH levels and younger age. Change in 27-OH was not significantly associated with the change in neuroimaging markers during 2 years. However, higher 27-OH at baseline was associated with lower total gray matter and hippocampal volume and lower cognitive test scores. These associations were mostly unaffected by total cholesterol levels. While sex seemed to impact most associations at baseline, it did not affect longitudinal associations. 27-OH should be further investigated not only as a potential biomarker for dementia/AD risk, but also as a potential marker for monitoring the effect of dementia preventive interventions.

## Data Availability

Data are available upon request. Public deposition of the de-identified data set is not possible due to legal and ethical reasons, and complete de-identification is not possible as this investigation is part of an ongoing study. The study participants gave informed consent which includes data use only under a confidentiality agreement. Further, the data contains a large amount of sensitive information, and public data deposition may pose privacy concerns. Those fulfilling the requirements for viewing confidential data as required by the Finnish law and the Finnish National Institute for Health and Welfare are able to access the data after completion of the material transfer agreement. Requests may be addressed to kirjaamo@thl.fi. Shared data relevant to the present study will encompass the data dictionary and pseudonymized participant data only. Study protocol including statistical analysis plan for the FINGER trial has been previously published [31, 35].

## Abbreviations

27-OH: 27-Hydroxycholesterol
FINGER: Finnish Geriatric Intervention Study to Prevent Cognitive Impairment and Disability
AD: Alzheimer’s disease
WML: White matter lesions
NTB: Neuropsychological test battery
ApoE: Apolipoprotein E
MRI: Magnetic resonance imaging
PET: Positron emission tomography
PiB: C-Pittsburgh compound B
